# The Royal Infirmary, Liverpool

**Published:** 1906-06-16

**Authors:** Francis H. Moore

**Affiliations:** Gen. Supt. and Sec. Liverpool


### Editor's Letter-Box.

### THE ROYAL INFIRMARY, LIVERPOOL.

*To the Editor of* The Hospital.

Dear Sir,—Referring to the paragraph in your issue of the 2nd instant respecting our report, I beg to say the illustrations are no new thing, a group of views of the interior of the building having formed the frontispiece of the one for the year 1897, and, with occasional variations, they have been a feature since; in fact, I believe the example referred to initiated the practice which has now become so universal.

On the question of accounts, our treasurer does not see in what way economy would be effected or contributions induced by the publication of more details of our system of book-keeping. Our statements have the approval of an eminent firm of accountants, and we believe they satisfy those who support the institution.

From the tables published in your Annual it is evident that our expenditure for many years has compared very favourably with that of similar institutions in the provinces, not to mention the metropolitan ones, which are quite out of court in the matter; and this, we believe, is effected without the least loss of efficiency from undue parsimony. If in this light further details would be helpful to other institutions their inclusion in our report is certainly worth considering.

The fact is regrettable that the "King's Fund" exists for the benefit of the most extravagant hospitals, and it seems a pity it does not insist on a more effective remedy for that condition of things than the adoption of an elaborate system of book-keeping.

With an experience of hospital management exceeding thirty years (partly acquired at a metropolitan one), I have learnt that although accounts may show where extravagance exists, the control of it is quite another matter, and the recent essays which should have made the path clear are no help in that direction.

Yours truly,

Francis H. Moore,

Liverpool, June 7. Gen. Supt. and Sec.

. ·. It is only possible in the case of institutions which keep their books upon the Uniform System to make an accurate and satisfactory comparison of the economical working and expenditure under all heads. At the present time the index of classification which forms an essential part of the Uniform System of accounts is being revised and brought up to date by a committee, so that the completeness of all comparisons as to cost may in future be made as full as possible. The question of hospital accounts does not merely concern individual institutions. For the larger hospitals which adopt the Uniform System not only contribute to the efficiency of their own work, but render an important service to hospital economy throughout the country. We are glad to see from the third paragraph of Mr. Moore's letter, that he appears to be alive to this fact, and we still hope that the Treasurer and himself may use their influence to ensure the early adoption of the Uniform System at the Royal Infirmary, Liverpool.—Ed. The Hospital.

